# The Mite-Gallery Unit: A New Concept for Describing Scabies through Entodermoscopy

**DOI:** 10.3390/tropicalmed4010048

**Published:** 2019-03-16

**Authors:** Gaetano Scanni

**Affiliations:** MD Dermatologist, Dipartimento di Medicina dei Servizi ASL Bari, Via Vico Traversa 11, 70127 Bari, Italy; gaetano.scanni@alice.it

**Keywords:** Mite-Gallery Unit (MGU), Entodermoscopy (EDS), Dry Dermatoscopy (d-DS), Wet Dermatoscopy (w-DS), Enhanced Dermatoscopy (e-DS)

## Abstract

Scabies has always represented a diagnostic challenge for dermatologists, especially in subclinical cases or in atypical ones due to the coexistence of other diseases. Fortunately, dermatoscopy has enabled easier and faster in situ diagnosis. The aim of this study is to examine old and new dermatoscopic signs that *Sarcoptes scabiei* produces on the skin during its whole life cycle through *entodermoscopy* (dermatoscopy with an entomological focus) which, unlike traditional optical microscope examination, allows the local micro-environment to be preserved intact. Patients were enrolled during outbreaks of scabies from hospitals or nursing homes for the elderly in Bari (Italy). The study was performed applying both immersion and polarized dry dermatoscopy. The systematic use of dermatoscopy highlighted the morphological complexity of the *Sarcoptes* tunnel that had been described previously as a simple unitary structure. On the contrary, it is possible to distinguish three separate segments of the burrow that introduce a new anatomo-functional concept called the Mite-Gallery Unit (MGU). This approach, based on the mite life cycle and local skin turnover (the latter usually being ignored), allows the dermatologist to recognize not only *Sarcoptes* using the gallery, but also new descriptors including tunnels without *Sarcoptes*, those with acari alone, and those with associated signs of inflammation. The diagnosis of scabies using optical microscopy until recently has always involved demonstrating the mite and its products outside the human body (on a glass slide) without taking into account exactly what happens within the epidermis. Entodermoscopy is a term used to encapsulate both the presence of the parasite, the usual target of microscopy, and the changes produced in the superficial layers of the epidermis in situ. Thus, the scabies tunnel or burrow can be shown to be composed of three parts, the Head, Body, and Tail, in which different events affecting both mite and host develop. The Mite-Gallery Unit provides a new anatomical and functional explanation of scabies because it provides a more comprehensive in vivo and in situ dermatoscopic diagnosis. In this respect, dermatoscopy takes into account the behavior of the mite in addition to its interaction with its habitat, the human skin.

## 1. Introduction

Diagnosis of scabies, even in the presence of objective and subjective epidemiological evidence, requires the detection of mites and their products. In clinical variants or in forms complicated by co-morbidities (diabetes, senile pruritus, neurological syndromes) or side effects of therapy (cortisone, acaricides), the detection of mites is absolutely essential for diagnosis because other clinical clues are unreliable or misleading [1]. The purpose of this article is to demonstrate that, by using entodermoscopy, it is possible to obtain direct and indirect evidence which may improve diagnostic reliability in both ordinary and atypical cases of scabies.

## 2. Materials and Methods

Two types of dermatoscope were used. The Delta 20 (Heine Optotechnik GmbH & Co. KG Kientalstraße 7, 82211 Herrsching, Germany) uses a glass plate and mineral oil at the skin interface in a mode that will be referred to as wet dermatoscopy (w-DS). The DermLite Pro II (Dermlite, 3Gen, San Juan Capistrano, CA, USA) is a dermatoscope without plate and liquid as it uses a polarized light that blocks the reflections from the epidermis; a side button allows the operator to switch alternately between polarized and normal light. This mode of observation will be referred to as dry dermatoscopy (d-DS). Pictures were obtained using two compact digital cameras: a Minolta G850 (5 Mp 3× optical, Minolta Corp., Osaka, Japan) for the Delta 20 (w-DS) and a Sony W70 (7 Mp 3× optical, Sony Corp., Tokyo, Japan) for the Dermlite (d-DS). The method using a dermatoscope with a digital camera is commonly referred to as digital dermatoscopy. The clinical observations herein are based on 63 patients examined in nursing homes for the elderly or public hospitals in the city of Bari (Italy) during contained outbreaks of scabies over the period 2011–2014.

Photographic documentation was generated after receiving patients’ informed consent.

### Entodermoscopy as a Diagnostic Tool

Entodermoscopy is a neologism proposed in 2006 by Scanni and Bonifazi for the dermoscopic diagnosis of ectoparasitosis [2], subsequently extended to superficial skin infections by Zalaudek et al. [3]. Entodermoscopy is not only a new term, but, importantly, it represents a different approach to the observation of the relationship between host and parasite for which a deeper knowledge of microbiology, entomology, and behaviour is required. Epiluminescence microscopy can be compared to traditional microscopic examination in which the instrument is resting on the patient to look directly at the skin. Consequently, the mite and its burrow can be observed in situ and in vivo, two conditions that together allow the observer to preserve the local microhabitat which is disassembled by more traditional methods (e.g., optical microscopy). As a tool, entodermoscopy has two major requirements. First, it requires magnifications higher than 10×, which can be achieved in digital dermatoscopy by extending the camera’s optical zoom (generally about 3–5×) and in post-production by examining the image (jpg file) in full format. Thanks to the combination of these two steps (maximum optical zoom + full-frame jpg file), sufficient detail is obtained to capture all the features of the tunnel in a mode that can be referred to as enhanced digital dermatoscopy (e-DS). This approach can help the dermatologist when videodermatoscopy is not available. Digital zoom is generally avoided because artifacts (pixelation/noise) are incompatible with a true and reliable image. The second requirement of entodermoscopy is the type of illumination and mode of contact between instrument and skin. The differences between wet contact and dry non-contact dermatoscopy in the case of melanocytic lesions are already known [4]. In the case of parasitosis, polarized dermatoscopes offer the advantage of leaving the microhabitat intact without the need to wet or crush the field of observation. This option is essential with, for example, pathogens such as lice or ticks.

In addition, the alternate switching from normal to polarized light allows a quick comparison between skin texture and structures below it, respectively, as well as the insect’s cuticle and internal organs, accurately completing the morphological and topographical reconstruction. The new findings described in this article derive from the comparative use of different dermatoscopes and light sources.

## 3. Results

In health facilities where scabies has already been diagnosed and treated, the main objective is often to understand if persistent itching is caused by the side effects of acaricides (e.g., benzyl benzoate), pre-existing co-morbidities, failure of therapy, or re-infestation. Since direct examination is not always sufficient to resolve these questions and optical microscopy is time consuming, any other instrumental help, including dermatoscopy, is worthy of attention and investigation. The methodical use of dermatoscopes has led to the introduction of a list of eight points, some already known and others previously undescribed, as an innovative morphological/functional key.

### 3.1. Definition of the Mite-Gallery Unit (MGU)

**Figure 1:**
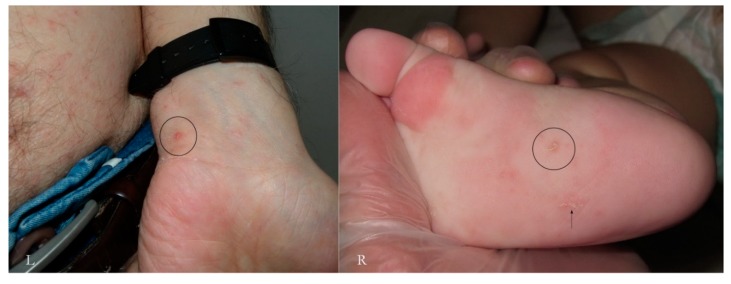
Objective examination of scabies in adults (L) and children (R).

Generally, the burrow of scabies is described as a linear formation a few millimeters in length that has always been considered a unitary structure (Figure 1).

An intact burrow is a whitish, slightly raised linear structure with a tortuous path a few millimeters long set against a variably erythematous background (circles, L-R). The appearance is pathognomonic. However, scratching that partially or completely destroys it can change its macroscopic and dermatoscopic appearance (arrow, R).

**Figure 2:**
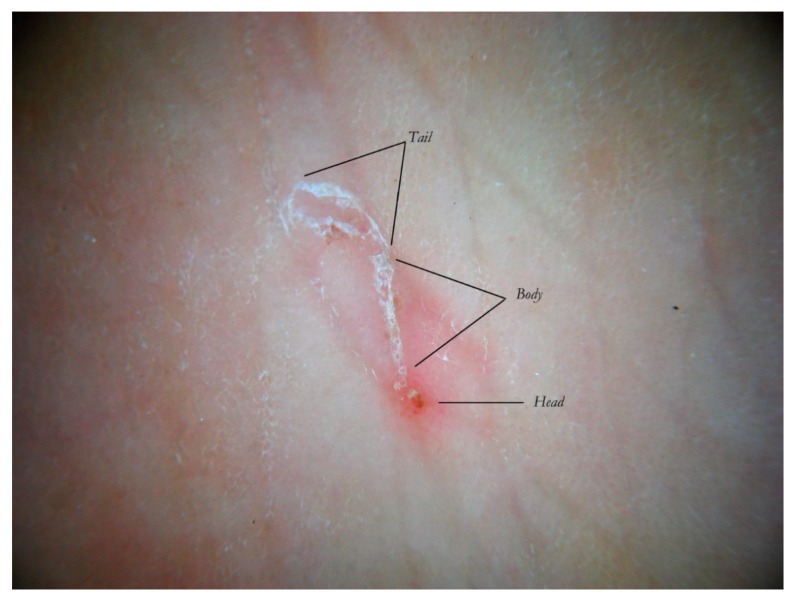
The Mite-Gallery Unit (MGU) in non-polarized dry dermatoscopy (d-DS).

Dermatoscopy reveals different *anatomical and functional* sections determined by both the mite life cycle and host epidermal turnover usually not taken into consideration but, none the less, able to affect the morphology of the tunnel itself. The gallery can be divided into three contiguous segments—Head, Body, and Tail—that together provide a new set of diagnostic markers called the Mite-Gallery Unit (MGU) (Figure 2).

The Mite-Gallery Unit (MGU) structure can be divided into three parts: the *Head* hosting the mite; the *Body*, which represents what is clinically defined as the burrow containing the eggs and feces of the parasite; and the *Tail* at the end of the tunnel, which provides an incomplete structure as it is without a roof but is made of keratin collarettes, visible only in d-DS. Erythema may be present in the background around and immediately behind the mite.

The dermatoscopic observation should preferably be conducted at magnifications between 10 and 30× with different results depending on whether a liquid interface (wet dermatoscopy) or dry skin surface free from the compression of the plate (dry dermatoscopy) are used.

The MGU Head houses the mite which is visible due to its refractile area located anteriorly between the buccal apparatus (*gnatosoma*) and the second pair of legs. It appears as a dark “V” formation with the vertex that indicates the point of progression of the tunnel towards the healthy skin. In this segment, the roof of the tunnel is intact.

The MGU Body represents the longest part of the entire tunnel and appears white if observed with wet dermatoscopy. In this segment, the roof of the tunnel is no longer intact because it is interrupted by holes at roughly regular intervals.

The MGU Tail is the terminal segment of the tunnel even if the roof is completely absent. This part can only be identified in dry dermatoscopy as this is able to define the edges of the tunnel in the form of small desquamatory collarettes. In w-DS the liquid medium prevents these from being visible, which explains why in previous studies this morphological variation was not reported.

The first description of the tunnel compared its appearance to that of a jet plane with delta wings followed by a white trail as a result of observation under wet dermatoscopy [5]. The observation of this phenomenon, however, was not accompanied by any functional interpretation. Entodermoscopy by studying the microstructure of the three components of the tunnel explains the “jet trail” effect as the interaction between the liquid interface and air contained in the tunnel that, due to the pressure of the dermatoscope, emerges from the small holes of the roof, forming a strip of small reflective bubbles under the optical plate.

**Figure 3:**
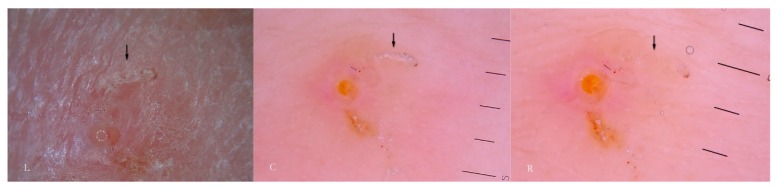
Dry dermatoscopy vs. wet dermatoscopy (w-DS) of the same Mite-Gallery Unit.

In fact, if dry dermatoscopy is used, the white trail is no longer visible and only a tunnel can be observed whose roof is interrupted by several openings from which air escapes if compressed (Figure 3).

When viewed using d-DS, the roof of the tunnel appears to be interrupted by several holes that allow air to exit (L). The first description of an MGU resembling a white jet trail was due to trapped air between the tunnel and the glass plate used in the w-DS (C). Under continued pressure, this picture disappears as all of air bubbles are pushed away and the liquid medium passes inside (R). Beneath there is a collection of serum that reflects the LEDs of the dermatoscope (L).

Another consequence of these holes, not previously described in the literature, is that the microbubble trail is by no means a persistent sign as it disappears when MGU observation is repeated several times or for excessive time in the wet modality. After the first contact with the dermatoscope, all the air inside the tunnel will be largely dispersed on the surface and replaced by the interface fluid; this makes the roof indistinguishable from the surrounding skin (Figure 3 R). In this situation, the tunnel body can only be recognized because of the head that houses the *Sarcoptes*, reducing the local diagnostic sensitivity which relies on the tunnel’s refractability. Therefore, in order to obtain good photographic documentation in w-DS of an intact MGU, it is necessary to keep the microbubbles under the plate without breaking off skin contact and to avoid repeating the viewing session. However, if air leakage decreases the dermatoscopic sensitivity as regards the external structure of the MGU, it provides ideal conditions to better observe the internal components of the tunnel (local diagnostic specificity).

### 3.2. Content of the Gallery and Other Markers of the Mite

**Figure 4:**
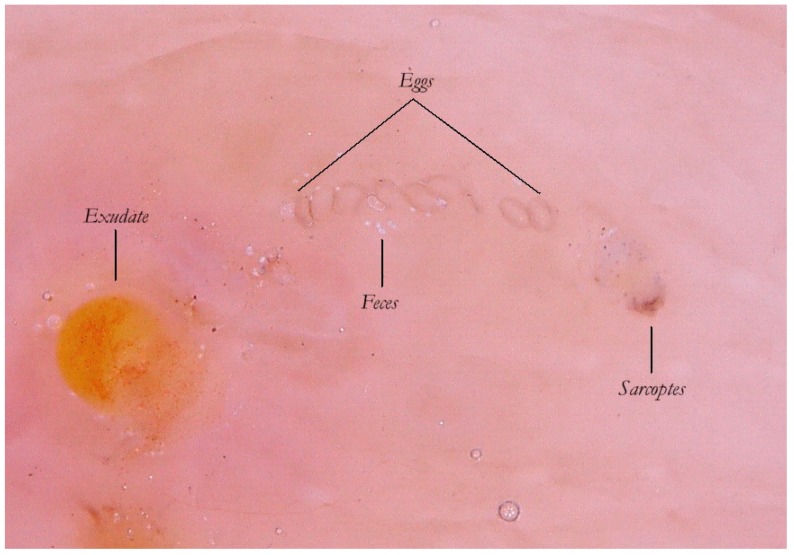
Content of the gallery. An MGU in enhanced wet dermatoscopy mode.

Thanks to the higher magnifications (e-DS) and to the fact that the roof of the tunnel in w-DS becomes transparent when liquid medium is inside, it is possible to distinguish, with sufficient clarity, the eggs of the *Sarcoptes* located behind the mite. The oval eggs are grouped at a short distance from each other with a major axis that is generally at right angles to that of the MGU, which also allows the edges of the embryo inside to be glimpsed, if enlarged further. In this part of the gallery there are also the parasite’s feces derived by the digestion of keratin and cellular fluids; under the optical microscope, feces usually appear as dark refractile dots (stercoraceous bullets), whereas under dermatoscopy, they appear as small white-gray spheres (Figure 4).

The absence of bubbles and the penetration of the liquid film into the tunnel makes the roof transparent and allows one to glimpse the row of eggs, which is otherwise not easily visible. The white dots are the feces of the mite. A seropurulent exudate is located to the left.

If the examiner is interested in studying the inside of the tunnel, he/she must use wet dermatoscopy and make sure that all the air in the tunnel is extruded, pushing the dermatoscope plate several times onto the skin to ensure entry of the liquid medium.

**Figure 5:**
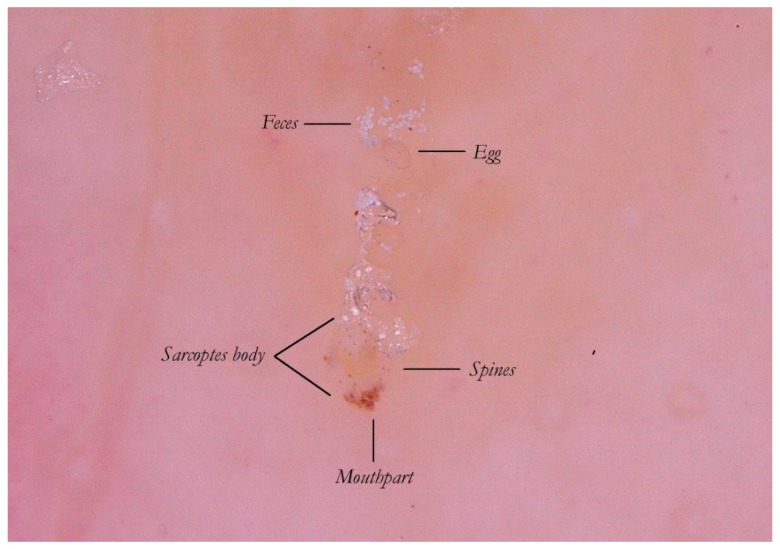
The Body of the mite. Enhanced wet dermatoscopy of an MGU in w-DS mode. In addition to refraction of its anterior part, *Sarcoptes scabiei* shows an opalescent body with several scattered dark dots (*ladybird sign*). These structures correspond to the “bristles” on the body that enable, amongst other things, the adherence of the mite within the tunnel.

It is commonly thought that the only visible part of the mite in w-DS is the front end, in the shape of a “V” which corresponds to the *gnatosoma* and the first pair of legs. But enhanced dermatoscopy allows the identification of novel structures on the body of the *Sarcoptes scabiei* that, under conditions of optimal magnification, show some visible refraction. These are mechano-sensory structures [6] located on the back of the mite called “bristles” (*spine-like type*) whose thicker bases (*sockets*) appear under a dermatoscope as fine brown dots (*ladybird sign*). This feature, not yet reported in the literature, is a good example of convergence between dermatoscopy and entomology. Even the body of the mite, which is usually transparent, can be distinguished by the slight opalescence of its cuticle under enhanced wet dermatoscopy (Figure 5).

### 3.3. Spatio-Temporal Evolution of the Mite-Gallery Unit

The MGU is not a static structure. This concept is not usually explored further because it is irrelevant to clinical diagnosis. On the contrary, patterns change if we access the microscopic details that are appreciable only in situ and in vivo in the context of the entodermoscopy.

The MGU is a dynamic structure because its morphology derives from a reciprocal interference of two different movements: the horizontal one of the mite and the vertical one of the epidermis. The epidermis has an estimated turnover time of around 45 days [7] during which the cellular layers are pushed upwards, ending with desquamation. In the presence of local inflammation (e.g., psoriasis) the upwards growth rate is greatly accelerated.

Histologically, the scabies burrow was always thought to be allocated in the *stratum corneum* even though confocal microscopy has recently revealed that the *Sarcoptes* mite is located closer to the *stratum spinosum* [8].

The integrity of the tunnel is therefore subordinate to the mite’s ability to maintain the same height in the epidermis to resist the tangential (rubbing) and elastic (contraction and distension) forces that act on the skin during the natural movements of the human body. The mite must out of necessity compensate for the direction of growth of the epidermis [9] in order to protected itself, but especially to ensure that the eggs can hatch just inside the tunnel. It follows that the tunnel represents two different time sequences depending on the point being observed, with the head being the newest part and the tail the initial and oldest part.

Therefore, as a dynamic structure the gallery moves closer to the skin surface with the passing of time and is at its most superficial at the tail where the roof becomes thin enough to collapse under the effect of the forces acting on it [10]. For this reason, near the tail there is no longer an intact tunnel but, rather, the borders of its base as collarettes visible only with dry dermatoscopy. Entodermoscopy, through linking the local factors of the host to the biology of the parasite, highlights an anatomic and functional difference of the MGU not previously appreciated under conditions of ordinary optical microscopy which cannot provide a detailed view of the mite’s whole microenvironment. Dermatoscopy instead reveals that the MGU is a dynamic entity in terms of both space (elongation phase) and time (maturation phase).

The collapsed roof is the result of an autonomous phenomenon and cannot be attributed to the scratching by the host as that would also destroy the other parts of the gallery rather than a very small part of it (the tail). This is further confirmed by the fact that the skin immediately around and adjacent to the tail remains intact (Figure 2).

### 3.4. MGU Perforated Roof

**Figure 6:**
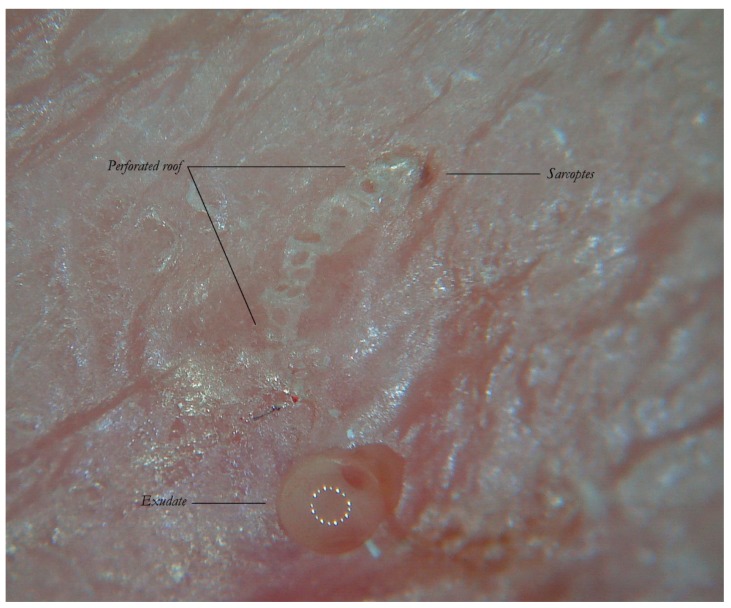
Enhanced dermatoscopy of the MGU’s roof in non-polarized dry mode (d-DS). The body of the gallery is not completely intact due to numerous holes which give it a riddled aspect. From these openings the larvae of the *Sarcoptes* are believed to exit. On the right, one can see the reflective front part of the mite. At the bottom, dermatoscope LEDs are reflected on the serous exudate.

As mentioned above, the roof of the MGU body is interrupted by holes (Figure 6). At the moment, the reason for these openings has not been precisely identified [11]. It is possible that they represent the exit sites for the larvae to leave the tunnel, but other explanations cannot be ruled out. The possibility exists that the *Sarcoptes* mite itself pierces the roof to guarantee exchange of air and humidity necessary for hatching the eggs. Another possibility is that the tunnel intercepts the ostia of the sweat glands by separating them from the rest of the duct. In any case, these holes cause the strip of microbubbles under the plate used in wet dermatoscopy to show the pathognomonic appearance of a “jet trail” [5].

### 3.5. Moulting and Coupling Pockets

The greater part of the MGU consists of the tunnel body where the most important events of the mite life cycle take place. In this segment, eggs are collected and hatch. After about four days, these eggs produce six-legged larvae morphologically similar to a miniature adult [12]. The larvae must undergo two more moults to the eight legged stage (proto/trito nymph) before becoming an adult; this process appears to take place mainly outside the mother tunnel in small niches, called “moulting pockets”, dug in order to provide limited shelter. Only when fully developed and after fertilization does the female mite dig a new definitive tunnel as it is known in temperate climates [13].

The moulting pockets, well known to entomologists, present as small dimples in the most superficial layer of the *stratum corneum*; in these, the larvae remain attached, maturing into nymphs and then into the adult [12].

**Figure 7:**
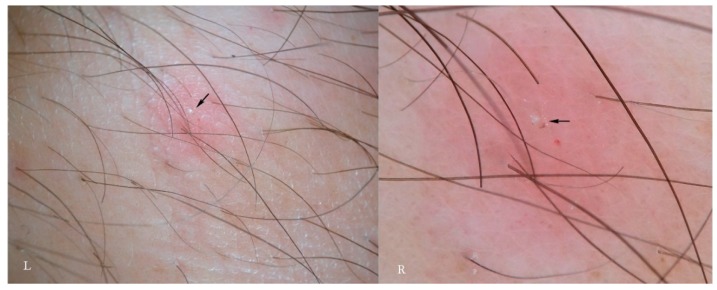
Moulting pocket in dry dermatoscopy with non-polarized (L) and polarized light (R). In this structure the complete Mite-Gallery Unit does not develop because the mite remains stationary until the moulting process is completed or mating happens. There is instead only a small whitish raised area (arrows, L-R), corresponding to the whole body of the *Sarcoptes*, behind which a real tunnel is not visible (R). Around the pocket, an area of erythema with blurred borders is easily recognizable.

Mating is also performed in a pocket in which the adult female is found by a male whose track is lost afterwards. Normally, these niches are not described in dermatological textbooks because they have no diagnostic relevance and are of dimensions invisible to the naked eye. However, their significance increases if we return to dermatoscopic examination in which they appear as an incomplete MGU composed only of a head without a body and a tail (Figure 7), regardless of the dermatoscope used. The preferred mode of observation is still the d-DS with normal or polarized light.

**Figure 8:**
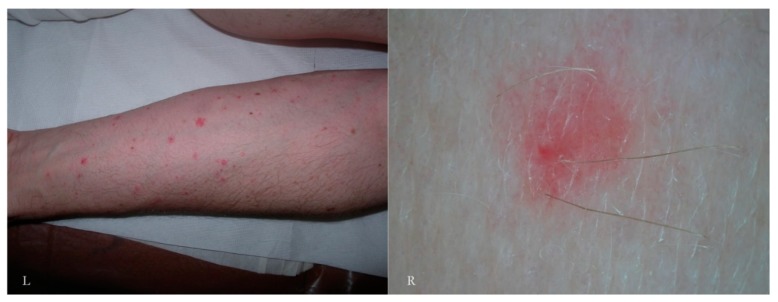
Scabies pseudo-follicular exanthema observed in non-polarized dry dermatoscopy. Folliculitis-like lesions (L) are commonly considered to be a non-specific sign of scabies (L). In dermatoscopy, only a peri-ostial erythematous halo is appreciated with some pinpoint-like vessels inside it (R). There are no signs of a complete or atypical MGU.

According to another report, the immature forms, besides digging a niche or pocket from scratch, can also take refuge in the openings of hair follicles [14]. A rash of pseudo-folliculitis is traditionally considered to be a secondary, non-specific symptom in the course of scabies (Figure 8). Assuming that the juvenile mite may shelter in human follicles, too, then the erythematous change associated with them may be regarded as a new “specific” sign. At the moment, personal observations and those of other authors have not been able to identify any traces of immature forms as features of in vivo refraction in these forms are unknown. It is possible that under higher magnification (>200×) these forms may become appreciable if present and visible.

### 3.6. Inflammatory Response Induced By the MGU

In a primary infestation, the host’s immunity to scabies develops after about four weeks. If we consider only the skin around the MGU, using dermatoscopy we can distinguish different types of response over time and depending on the anatomical sites involved. Generally, there is erythema around the head of the gallery where the mite is located or along the entire length of the MGU.

**Figure 9:**
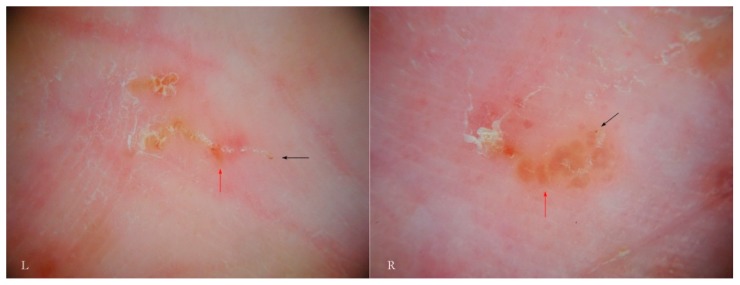
Inflammatory response to an MGU observed under polarized light dry dermatoscopy of a hand lesion. Next to the mite (black arrows, L-R) the skin is normal, while in the central part of the gallery an erythematous halo (red arrow, L) is evident. Immediately behind the mite, inflammation can also occur in microvesicles trapped in the epidermis (red arrow, R). The tail of the gallery is characterized by keratin collarettes.

A vesicular or free exudate appears when the spongiotic inflammatory process [15] reaches the skin surface. If an infection coexists, this phenomenon is more pronounced. Where the skin is thicker, as on the palm of the hand and on the wrist, the exudate remains trapped in the superficial epidermis, forming semitransparent (*dyshidrotic-like*) circular areas [16] that, over time, become superficial as yellowish desquamations (Figure 9). The mite, the feces, and the eggs activate a different humoral and cellular immune response, probably depending on the time of exposure to the antigens that gradually accumulate over the course of the infestation. This reflects the life cycle of the mite.

**Figure 10:**
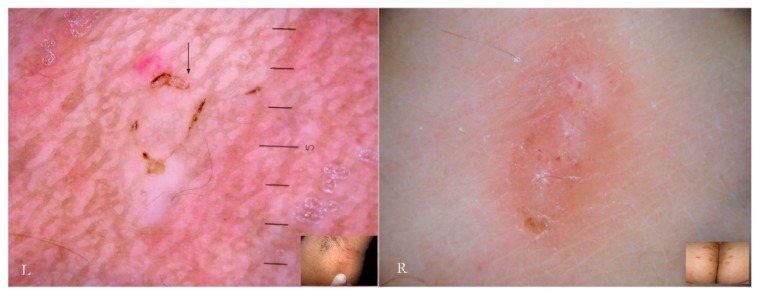
Nodular scabies observed in wet (L) and dry polarized light (R) dermatoscopy. A mite is easily recognizable in one of the axillary (insert) papules of a subject of African ethnicity (arrow, L). A buttock (insert) nodule present for a few weeks appears uninhabited instead (R).

In other cases, the inflammatory response may be nodular as in the buttocks, genital, and flexural regions. This phenomenon, known for its resistant pruritic symptoms, is interpreted as the effect of cell-mediated hyperreactivity to mite antigens [17] even if scrapings for mites are generally negative. Dermatoscopy, however, can identify the parasite, especially in the initial phases of the formation of nodules compared to the older lesions (Figure 10). Serial dermatoscopic observations could clarify the exact progression of the host’s local immune response, probably stimulated by deeper penetration of antigens (intradermal) in areas undergoing physical pressure.

### 3.7. Morphological Variants of the MGU

**Figure 11:**
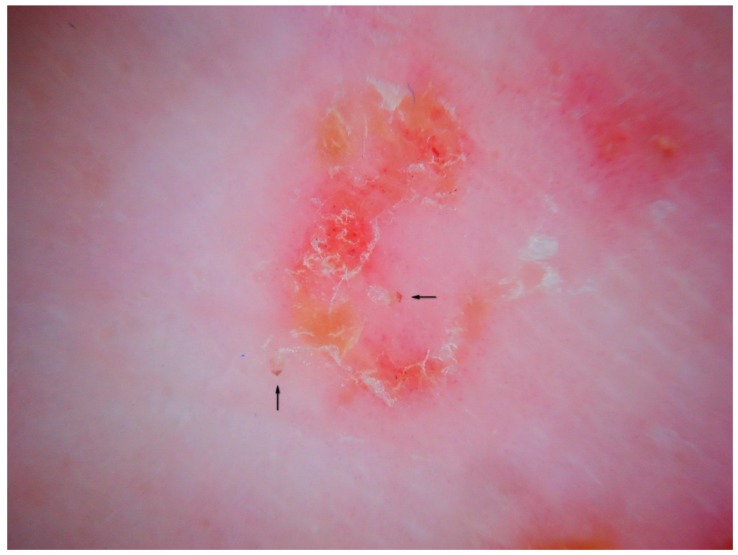
Dry dermatoscopy with polarized light of an atypical MGU. Two mites (arrows) can be recognized behind which there is no tunnel but an erythematous area with polycyclic borders.

**Figure 12:**
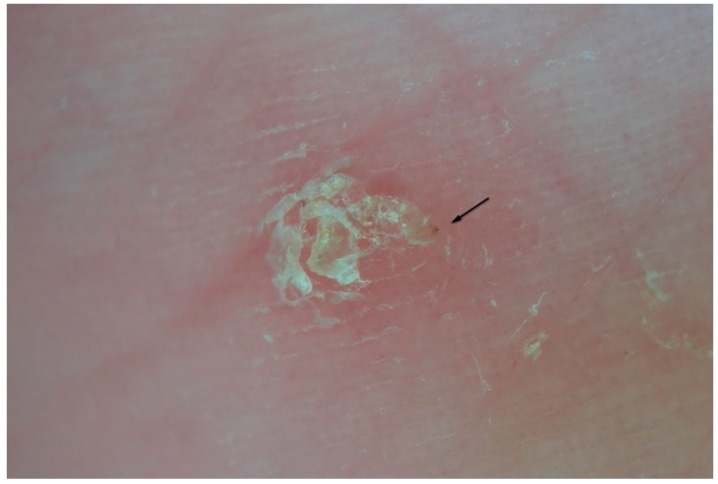
Dry dermatoscopy with non-polarized light of an atypical MGU. The refractive part of the mite (arrow) is evident, behind which there is an aggregate of scales rather than an ordinary gallery.

The linear MGU is not the only expression of the mite in the host skin–parasite interaction. There are forms of “atypical MGUs” in which only the head of the gallery is recognizable but not the body because it is partly or completely replaced by erosions or polycyclic scales (Figure 11 and Figure 12). These morphological variants develop in areas where local exudative or hyperkeratotic inflammatory reactions have occurred; this fact must be kept in mind because, although they appear clinically to be secondary lesions, this can be excluded by the dermatoscopic examination. The preferred mode of observation is dry dermatoscopy because it is more sensitive to the surface texture. In fact, as it misses the tunnel, wet dermatoscopy would not show any trail of bubbles and the wet scales could become slightly refractive.

### 3.8. MGU Evolution at the End of the Mite Life Cycle or After Therapy

**Figure 13:**
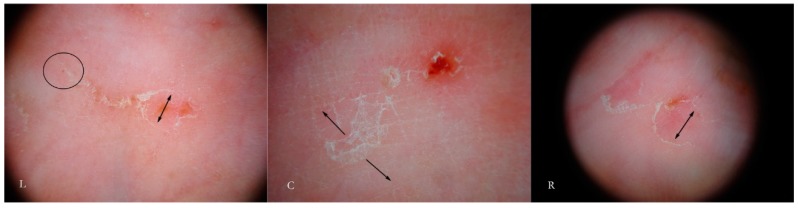
Evolution of an MGU towards a “ghost gallery” under polarized dry dermatoscopy. Three different examples. A mature MGU ends with a tail made up of keratinised collarettes that progressively move away from each other (black arrow, L). *Sarcoptes* is identifiable in the front part of the burrow (circle, L). When the mite is at the end of its life cycle or after therapy, the other parts of the tunnel undergo the normal processes of remodelling of the skin (C), forming the thin and polycyclic keratinic edges of a ghost gallery (C/R).

When the formation of new parts of the tunnel ceases, due to therapy or termination of the mite’s life cycle, after a few days, all the MGUs will be remodeled and replaced only by traces in the form of polycyclic keratinised outlines appearing like a “*ghost gallery*”. Post-inflammatory phenomena and local superinfection can also contribute to their formation. Detection of phantom galleries might be useful in assessing scabies when the therapeutic outcome is uncertain, or in the absence of active MGUs, to confirm the success of a therapy, itching being a subjectively variable criterion (Figure 13).

## 4. Discussion and Conclusions

Since dermatoscopy provides the description of the only pathognomonic sign of scabies (the burrow) as “delta wing plane + jet trail” [5], dermatologists have tried to replace the traditional scraping test with a faster dermatoscopic diagnosis which is more patient friendly [18].

The mite together with its tunnel is an important high diagnostic feature, but the global diagnostic sensitivity remains low because burrows, during clinical examination, are visible in only about 20% of patients [19]. In this article, the effect of *Sarcoptes* on and within the skin is described as an anatomical and functional entity, that is dynamic in both space and time. This is called the Mite-Gallery Unit (MGU), which consists of a head, body, and tail that take on a definable role in the production of the earlier and later dermatoscopic semeiotics of the insect’s life cycle.

This setting allows us to add new diagnostic descriptors in which *Sarcoptes* can be recognized in exudative, hyperkeratotic, and nodular lesions clinically considered to be non-specific but which, on the contrary, are useful sites for dermatoscopic examination.

To detect these signs, it is necessary to use both dry polarized and wet dermatoscopy helped by specific maneuvers to make the outside and inside of the tunnel visible (local diagnostic specificity).

Thanks to entodermoscopy, two other parts of the mite (spines and body cuticle) can be identified, making the diagnosis more reliable even when the *acarus* is not associated with a visible tunnel. The juvenile forms hitherto excluded from the diagnosis can be detected in superficial epidermal *pockets* whose pathological effects are still unknown. Considering human skin turnover is an integral element in the diagnosis it is possible to follow the fate of the MGU even when, at the end of its life cycle, the mite no longer forms new parts of the tunnel but a “phantom” imprint remains nonetheless.

No traditional microscopic examination has ever produced a detailed anatomical and functional description of the tunnel such as the one obtained via entodermoscopy. The principles and rationale of entodermoscopy are very close to a behavioural approach as they involve the study of the acari, while taking care not to modify the local habitat—something that is a feature of traditional optical microscope examinations in scabies and other ectoparasitoses.

A new instrument (*entodermoscope*) with higher magnification (>200×), different light source options (normal/polarized/tangential), and micromanipulators operating in the field of examination could add valuable information as yet unknown. Meanwhile, entodermoscopy provides a new vision of the host–parasite relationship which will benefit diagnosis and as seems likely, in the future, our understanding of transmission and the assessment of innovative preventive or therapeutic strategies.
